# Conservation of a crystallographic interface suggests a role for β-sheet augmentation in influenza virus NS1 multifunctionality

**DOI:** 10.1107/S1744309111019312

**Published:** 2011-07-13

**Authors:** Philip S. Kerry, Elizabeth Long, Margaret A. Taylor, Rupert J. M. Russell

**Affiliations:** a Biomedical Sciences Research Complex, University of St Andrews, St Andrews, Fife KY16 9ST, Scotland

**Keywords:** effector domains, influenza virus, virulence factors, NS1, β-sheet augmentation

## Abstract

The effector domain (ED) of the influenza virus virulence factor NS1 is capable of interaction with a variety of cellular and viral targets, although regulation of these events is poorly understood. Introduction of a W187A mutation into the ED abolishes dimer formation; however, strand–strand interactions between mutant NS1 ED monomers have been observed in two previous crystal forms. A new condition for crystallization of this protein [0.1 *M* Bis-Tris pH 6.0, 0.2 *M* NaCl, 22%(*w*/*v*) PEG 3350, 20 m*M* xylitol] was discovered using the hanging-drop vapour-diffusion method. Diffraction data extending to 1.8 Å resolution were collected from a crystal grown in the presence of 40 m*M* thieno[2,3-­*b*]pyridin-2-ylmethanol. It was observed that there is conservation of the strand–strand interface in crystals of this monomeric NS1 ED in three different space groups. This observation, coupled with conformational changes in the interface region, suggests a potential role for β-sheet augmentation in NS1 function.

## Abstract 2

The structure of a monomeric effector domain from influenza A virus NS1 is presented from diffraction data extending to 1.8 Å resolution. Comparison of this and other NS1 effector-domain structures shows conformational changes at a strand–strand packing interface, hinting at a role for β-strand augmentation in NS1 function.

## 1. Introduction

The NS1 protein of influenza virus is an important virulence factor and has been demonstrated to interact with a wide variety of viral and cellular biomolecules (Hale, Randall *et al.*, 2008 ▶). In particular, crystallographic structures have been obtained of the N-terminal RNA-binding domain (RBD) in complex with dsRNA (Cheng *et al.*, 2009 ▶) and of the C-terminal effector domain (ED) in complex with the F2F3 portion of the cellular processing and specificity factor CPSF30 (Das *et al.*, 2008 ▶) and with the iSH2 domain of the PI 3-­kinase regulatory subunit p85β (Hale, Kerry *et al.*, 2010 ▶). Furthermore, both domains form homodimers *in vitro* (Bornholdt & Prasad, 2006 ▶; Chien *et al.*, 1997 ▶; Hale, Barclay *et al.*, 2008 ▶; Xia *et al.*, 2009 ▶). While the conformation of the RBD dimer appears to be conserved, two forms of the ED dimer have been proposed: the strand–strand dimer and the helix–helix dimer (Hale, Barclay *et al.*, 2008 ▶; Xia *et al.*, 2009 ▶; Bornholdt & Prasad, 2006 ▶). The helix–helix dimer is present in all wild-type NS1 ED structures (Kerry *et al.*, 2011 ▶) and in the structure of full-length NS1 (Bornholdt & Prasad, 2008 ▶). In contrast, the canonical strand–strand dimer has only been observed in crystallographic contacts in a few NS1 ED structures obtained using NS1 from the A/Puerto Rico/8/34 (PR8) strain. The structure of the full-length NS1 protein from an H5N1 strain has also been reported to exhibit this interface, although in a somewhat distorted form, leading to claims that the strand–strand dimer is involved in NS1 oligomerization (Bornholdt & Prasad, 2008 ▶). The introduction of a W187A mutation into the ED induces a monomeric phenotype (Hale, Barclay *et al.*, 2008 ▶; Kerry *et al.*, 2011 ▶; Xia & Robertus, 2010 ▶) and, intriguingly, two crystal structures of the PR8 NS1 ED containing this mutation also exhibit this strand–strand interface.

Although the NS1 ED is known to interact with several viral and cellular factors, only two structures of NS1 in complex with another protein have been solved. Therefore, the sites of many of the other interactions are very poorly characterized. Recently, a new model for NS1 regulation was proposed in which formation of the ED helix–helix dimer regulates interactions between NS1 and other factors (Kerry *et al.*, 2011 ▶). In particular, it was observed that binding to CPSF30 and PI-3-kinase were both incompatible with ED dimerization and would require separation of the two monomers. At present no function has been ascribed to the dimerized ED, although disruption of this interface may interfere with binding to dsRNA (Kerry *et al.*, 2011 ▶; Wang *et al.*, 2002 ▶). However, while the role of the helix–helix interface in ED dimerization appears to be settled, potential roles for the strand–strand interface remain to be explored. Interestingly, formation of the helix–helix dimer leaves the strand–strand interface available for other intermolecular interactions, allowing the possibility of the formation of alternative β-sheet interactions.

In this paper, we report a third X-ray crystallographic structure of the PR8 NS1 ED containing the W187A mutation. This structure is highly homologous to the previous structures obtained using this construct and conservation of the strand–strand packing interface highlights a possible role for β-sheet augmentation in NS1 function.

## 2. Experimental

The construction of the pRSFDuet plasmid expressing a His_6_-tagged version of PR8 NS1 ED (W187A) (residues 73–230) has been described previously (Kerry *et al.*, 2011 ▶). The plasmid was transformed into *Escherichia coli* Rosetta (DE3) expression strain (Novagen) for protein expression. The transformed *E. coli* cells were inoculated into Luria–Bertani (LB) medium with 50 µg ml^−1^ kanamycin at 310 K. 1 m*M* isopropyl β-d-1-thiogalactopyranoside (IPTG) was added to induce protein expression when the optical density at 600 nm (OD_600_) of the culture reached 0.6. Cell culture continued for 16 h at 295 K before harvesting by centrifugation at 7000*g* for 20 min at 277 K. The harvested pellet was resuspended in 20 ml phosphate-buffered saline supplemented with 1 m*M* MgCl_2_, 20 µg ml^−1^ DNase (Sigma), 200 µg ml^−1^ lysozyme (Sigma) and protease-inhibitor cocktail tablets (two tablets per 20 ml; Roche Diagnostics) and incubated for 2 h at 295 K, after which the crude cell extract was centrifuged at 20 000*g* for 15 min at 277 K. The supernatant was then supplemented by the addition of 5 ml 2 *M* NaCl and 0.5 ml 250 m*M* imidazole before loading onto a 15 ml nickel column (GE Healthcare). Bound protein was eluted using 500 m*M* imidazole and then dialysed for 16 h at 295 K against 200 m*M* NaCl, 50 m*M* Tris pH 7.4, 1 m*M* dithiothreitol, during which time cleavage of the His_6_ tag was achieved by addition of 200 U tobacco etch virus protease (Invitrogen). Uncleaved protein was removed from the dialysed fraction by loading it onto a 15 ml nickel column. The flowthrough fraction was then pooled for gel filtration using a 120 ml HiLoad 16/60 Sephadex 75 column (GE Healthcare).

The purified PR8 NS1 ED (W187A) was pooled and concentrated to 8.5 mg ml^−1^ using a 5000 MWCO Vivaspin column (Sartorius). Prior to screening for novel crystallization conditions, 40 m*M* thieno[2,3-*b*]pyridin-2-ylmethanol and 2% dimethyl sulfoxide were added to the purified protein. Screening was performed using the sitting-drop vapour-diffusion method at 290 K with the commercial kits Clear Crystal Strategy 1 and 2, Structure Screen 1 and 2 (Molecular Dimensions) and Classics II and PEGs II (Qiagen). After one week, needle-shaped crystals were observed using Classics II con­dition No. 71 (0.2 *M* NaCl, 0.1 *M* Bis-Tris pH 6.5, 25% PEG 3350). This condition was selected for optimization by the hanging-drop vapour-diffusion method with crystallization drops made up of 1 µl purified protein solution and 1 µl reservoir solution. The optimal conditions for crystallization were found to be 0.2 *M* NaCl, 0.1 *M* Bis-Tris pH 6.0, 22% PEG 3350, 0.02 *M* xylitol. Crystals appeared after 4 d and reached their maximum size after 14 d.

Crystals were cryoprotected by transfer to a solution of 20%(*w*/*v*) xylitol in crystallization buffer before data collection at 100 K. Data were collected in-house (Rigaku–MSC MicroMax-007 HF X-ray generator and Saturn 944+ CCD detector). Data processing and scaling were performed using *HKL*-2000 (Otwinowski, 1993 ▶). The previously obtained structure of PR8 NS1 ED (W187A) (PDB entry 3o9q; Kerry *et al.*, 2011 ▶) was used for molecular replacement using *Phaser* (McCoy *et al.*, 2007 ▶) in the *PHENIX* package (Adams *et al.*, 2002 ▶). *PHENIX* and *Coot* (Emsley & Cowtan, 2004 ▶) were used to refine the model, which was validated with *MolProbity* (Chen *et al.*, 2010 ▶). Data-collection and refinement statistics are shown in Table 1 ▶. Figures were created using *PyMOL* (Schrödinger, 2010 ▶).

## 3. Results and discussion

**Figure 1:**
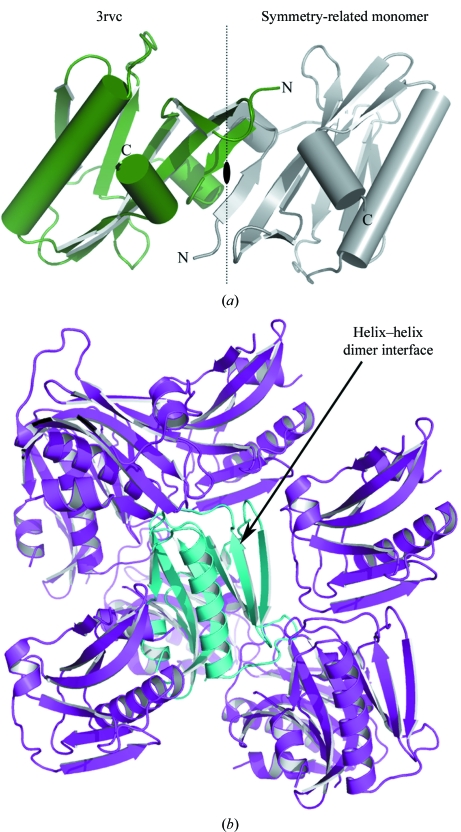
(*a*) Crystal structure of PR8 NS1 ED (W187A) described here (PDB entry 3rvc, shown in green). A strand–strand packing interface is formed with a symmetry-related molecule (shown in light grey). (*b*) Crystal-packing interactions formed between the PR8 NS1 ED (W187A) structure 3rvc (shown in cyan) and symmetry-related molecules (shown in magenta). The NS1 ED helix–helix dimer binding site is exposed.

The novel crystal form of PR8 NS1 ED (W187A) shows the α/β fold common to all structures of NS1 ED (Bornholdt & Prasad, 2006 ▶; Kerry *et al.*, 2011 ▶). In contrast to the majority of NS1 structures, only one molecule of PR8 NS1 ED (W187A) was present within the asymmetric unit, although a strand–strand interface homologous to those observed for previous structures obtained using this con­struct was observed between symmetry-related monomers (Fig. 1 ▶ *a*). As expected from its monomeric form, the helix–helix dimer present within all wild-type structures of NS1 was not observed in the crystal lattice (Fig. 1 ▶ *b*). This is in agreement with previous structures obtained using this construct, which also lack the helix–helix dimer interface (Kerry *et al.*, 2011 ▶). Therefore, since the PR8 NS1 ED W187A mutant is monomeric *in vitro* (Kerry *et al.*, 2011 ▶), it appears highly likely that the helix–helix dimer is the predominant interface for ED homodimerization. However, it is intriguing to observe that the strand–strand dimer is conserved among all three PR8 NS1 ED (W187A) structures that have now been solved. Additionally, this interface is also employed in two of the four wild-type PR8 NS1 ED structures [PDB entries 2gx9 (Bornholdt & Prasad, 2006 ▶) and 3o9u (Kerry *et al.*, 2011 ▶)]. This is a remarkable coincidence, especially considering that the crystals leading to these five structures belonged to different space groups (*P*3_2_21, *P*2_1_2_1_2_1_, *C*222_1_, *P*4_3_22 and *P*6_4_) and that conservation between mutant and wild-type structures is not observed for any other NS1 ED interface. Comparison of the strand–strand dimers indicates two distinct orientations of the monomers relative to one another (Supplementary Fig. 1^1^). Although one orientation predominates, the *AF* and *BD* dimers of 3o9u adopt a slightly twisted arrangement, indicating that there may be some flexibility in the contacts formed at this interface. The partial con­servation of the strand–strand packing interface indicates that while it may not allow ED dimerization, extension of the β-sheet is a possible method of interaction with cellular and viral binding partners. Interestingly, this interface is not observed in other structures of the NS1 ED, even when mutations preventing helix–helix dimerization are introduced [*e.g.* W187Y (PDB entry 3kwi) and W187A (PDB entry 3kwg); Xia & Robertus, 2010 ▶]. Therefore, it could be concluded that any functional properties of this interface may vary between influenza virus strains. Such strain-specificity has been observed for some functions of NS1, most notably interaction with CPSF30, which is associated with NS1s from H3N2 and H2N2 subtypes but not all isolates of the H1N1 subtype (Kuo *et al.*, 2010 ▶; Hale, Steel *et al.*, 2010 ▶). However, while the other NS1 ED solved from an H1N1 subtype (A/California/07/09; PDB entry 3m5r; Center for Structural Genomics of Infectious Diseases, unpublished work) does not exhibit a strand–strand dimer, it does show antiparallel strand–strand interactions *via* an alternative arrangement (Supplementary Fig. 2^1^). In structures of NS1 from PR8 the strand–strand dimer is formed by residues 88–91 of the two monomers interacting with one another to form a contiguous β-sheet; however, in the case of 3m5r residues 80–85 of chain *A* are sandwiched between residues 87–92 of chain *G* and residues 86–89 of chain *B*. This variant on the β-sheet augmentation theme suggests that such interactions may not be restricted by the sequences and arrangements observed in the strand–strand packing interface.

**Figure 2:**
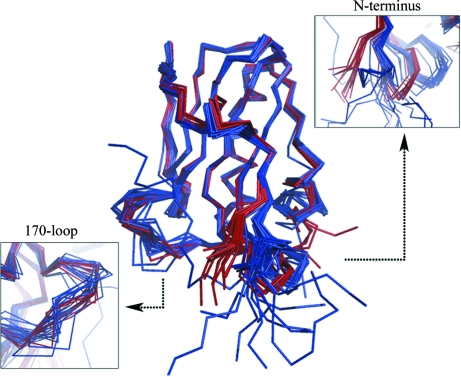
Superposition of monomers of NS1 ED. The monomers are aligned with PR8 NS1 ED structure 2gx9_*A*. Monomers participating in strand–strand interactions are coloured red, whilst those not participating in such interactions are coloured blue. Inserts highlight areas of increased variability within the NS1 ED structure: left, 170-loop; right, N-terminus and 140-loop.

Although the overall fold of the NS1 ED monomer is remarkably well conserved, comparison of all of the ED monomers solved to date indicates two regions of significant variation between the structures (Fig. 2 ▶). One region that appears to be capable of adopting a variety of positions is the 170-loop (residues 162–170), which is present at the interface between the ED and the iSH2 domain of p85β and has also been proposed to be a putative SH3-binding motif (Hale, Kerry *et al.*, 2010 ▶; Shin *et al.*, 2007 ▶). While the structure of this loop is always well ordered, the positions adopted vary between structures regardless of the strain or subtype (Fig. 2 ▶, left insert). Flexibility in this region may indicate a propensity for binding to a number of factors in addition to p85β, as several orientations may be required for different binding events.

A second region of variance between NS1 ED monomers which has been observed previously (Hale, Barclay *et al.*, 2008 ▶) is the N-­terminus of the ED (up to residue 91) and the β-hairpin loop (140-­loop) between the fourth and fifth β-strands (residues 135–143) (Fig. 2 ▶ *b*, right insert). These residues appear to occupy one of two conformations, for which the *A* chain of 2gx9 (Bornholdt & Prasad, 2006 ▶; 2gx9_*A*) and the *A* chain of 3d6r (Hale, Barclay *et al.*, 2008 ▶; 3d6r_*A*) could be considered to be archetypical structures. Interestingly, the position of this region does not appear to depend upon the sequence or strain of the ED crystallized, but rather on whether a strand–strand contact is formed within the crystal lattice. For example, the tertiary structure of the PR8 ED 3o9s_*A*, which does not form a strand–strand dimer, bears more similarity to 3d6r_*A* than to the previously characterized PR8 ED structure 2gx9_*A*. Furthermore, a comparison of each ED monomer solved to date with these two archetypes (2gx9_*A* and 3d6r_*A*) showed that while no monomer structure differed from either of the structures by more than 1.04 Å, all ED monomers involved in strand–strand contacts bore greater homology to 2gx9_*A* than to 3d6r_*A* (Table 2 ▶). Therefore, it appears to be likely that this orientation is induced by interactions at the strand–strand interface and may not exist outside of this context. In support of this view, examination of the NMR structure of an Udorn effector domain (PDB entry 2kkz; Aramini *et al.*, 2011 ▶) indicates that the 3d6r_*A* conformation is adopted in all NMR states, while the 2gx9_*A * conformation is not present. While the W187R mutation present within the monomer used to collect these NMR data is likely to disrupt ED dimerization, it is located within the helix–helix interface and is unlikely to influence any strand–strand interactions.

While it appears to be unlikely that the strand–strand dimer is the predominant ED homodimer, the partial conservation of this interface and the ability of contacts at this interface to induce conformational changes are interesting and may indicate that this surface may be utilized in other intermolecular interactions. Furthermore, the observation that other sequences can form a β-strand addition at this interface, as seen in 3m5r, suggests that such β-sheet augmentations are unlikely to be restricted to the formation of a strand–strand dimer. Therefore, it is possible to envisage similar interactions existing between NS1 and one or more of the wide variety of factors that it is known to bind to.

## Supplementary Material

PDB reference: NS1 effector domain with W187A mutation, 3rvc

Supplementary material file. DOI: 10.1107/S1744309111019312/hv5191sup1.pdf

## Figures and Tables

**Table 1:** Crystallographic summary Values in parentheses are for the highest resolution shell.

Protein	PR8 NS1 Δ72 W187A
Space group	*C*222_1_
Unit-cell parameters (Å)	*a* = 32.0, *b* = 102.8, *c* = 67.4
Maximum resolution (Å)	1.80 (1.83–1.80)
Unique reflections	9857
Completeness (%)	92.2 (52.4)
Mean *I*/σ(*I*)	50.1 (3.9)
Multiplicity	6.4
*R*_merge_ (%)	0.048 (0.419)
*V*_M_ (Å^3^ Da^−1^)	2.12
Refinement	
Protein atoms	918
Water atoms	81
Resolution range (Å)	18–1.8
*R*_cryst_ (%)	19.4
*R*_free_ (%)	24.9
Mean temperature factors (Å^2^)	
Protein	27.8
Waters	35.6
R.m.s.d. bond lengths (Å)	0.007
R.m.s.d. bond angles (°)	1.054
Ramachandran favoured/outliers (%)	98.3/0

**Table 2:** Analysis of NS1 ED-monomer homology Root-mean-square deviations (r.m.s.d.s) were calculated for each monomer with respect to two archetypes, 2gx9_*A* and 3d6r_*A*, using *PyMOL*. Values in bold indicate the lower r.m.s.d. relationship. Strain abbreviations are as follows: PR8, A/Puerto Rico/8/34; Alb/76, A/Duck/Albany/60/76; Ud/72, A/Udorn/72; VN/04, A/Viet Nam/1203/2004; Cal/04, A/California/07/2009.

Strain	Structure (PDB code_chain)	Strand–strand dimer	2gx9_*A* r.m.s.d. (Å)	3d6r_*A* r.m.s.d. (Å)
Wild-type NS1 ED				
PR8	2gx9_*A*	Yes	**0.00**	0.91
PR8	2gx9_*B*	Yes	**0.30**	0.87
PR8	3o9s_*A*	No	0.85	**0.73**
PR8	3o9s_*B*	No	0.85	**0.60**
PR8	3o9u_*A*	Yes	**0.57**	0.72
PR8	3o9u_*B*	Yes	**0.55**	0.63
PR8	3o9u_*C*	Yes	**0.56**	0.63
PR8	3o9u_*D*	Yes	**0.57**	0.72
PR8	3o9u_*E*	Yes	**0.55**	0.63
PR8	3o9u_*F*	Yes	**0.55**	0.63
PR8	3o9u_*G*	Yes	**0.57**	0.63
PR8	3o9u_*H*	Yes	**0.57**	0.63
PR8	3o9t_*A*	No	0.61	**0.58**
PR8	3o9t_*B*	No	0.59	**0.57**
Alb/76	3d6r_*A*	No	0.91	**0.00**
Alb/76	3d6r_*B*	No	0.98	**0.31**
Alb/76	3oa9_*A*	No	0.98	**0.37**
Alb/76	3oa9_*B*	No	0.81	**0.52**
Ud/72	3ee9_*A*	No	0.87	**0.52**
Ud/72	3ee9_*B*	No	0.63	**0.59**
Ud/72	3ee8_*A*	No	0.87	**0.52**
Ud/72	3ee8_*A*	No	0.84	**0.53**
VN/04	3f5t_*A*	No	1.04	**0.61**
Cal/07	3m5r_*A*	No	0.79	**0.46**
Cal/07	3m5r_*B*	No	0.69	**0.61**
Cal/07	3m5r_*D*	No	0.75	**0.55**
Cal/07	3m5r_*E*	No	0.67	**0.56**
Cal/07	3m5r_*F*	No	0.68	**0.56**
Cal/07	3m5r_*G*	No	0.71	**0.69**
Mutant NS1 ED				
PR8 (W187A)	3o9r_*A*	Yes	**0.59**	0.82
PR8 (W187A)	3o9r_*B*	Yes	**0.56**	0.79
PR8 (W187A)	3o9q_*A*	Yes	**0.56**	0.66
PR8 (W187A)	3o9q_*B*	Yes	**0.61**	0.64
PR8 (W187A)	3rvc_*A*	Yes	**0.49**	0.69
Ud/72 (W187A)	3kwg_*A*	No	0.92	**0.49**
Ud/72 (W187A)	3kwg_*B*	No	0.81	**0.43**
Ud/72 (W187Y)	3kwi_*A*	No	0.83	**0.59**
Ud/72 (W187Y)	3kwi_*B*	No	0.65	**0.51**
NS1 ED in complex				
PR8 (with p85β)	3l4q_*A*	No	1.04	**0.52**
PR8 (with p85β)	3l4q_*B*	No	1.05	**0.58**
Ud/72 (with CPSF30)	2rhk_*A*	No	0.91	**0.39**
Ud/72 (with CPSF30)	2rhk_*B*	No	0.96	**0.47**
NMR structures of NS1 domains				
Ud/72 (W187R)	2kkz_*A*	No	0.93	**0.82**

## Footnotes

1 Supplementary material has been deposited in the IUCr electronic archive (Reference: HV5191).
