# Supplementary material for: Conservation of a crystallographic interface suggests a role for β-sheet augmentation in influenza virus NS1 multifunctionality
Source: Acta Crystallogr Sect F Struct Biol Cryst Commun. 2011 Jul 13;67(Pt 8):858–61. doi: 10.1107/S1744309111019312 (PMC3151114; doi:10.1107/S1744309111019312)
Supplement: Supplementary file 1 [file f-67-00858-sup1.pdf]

Supplementary Material

A

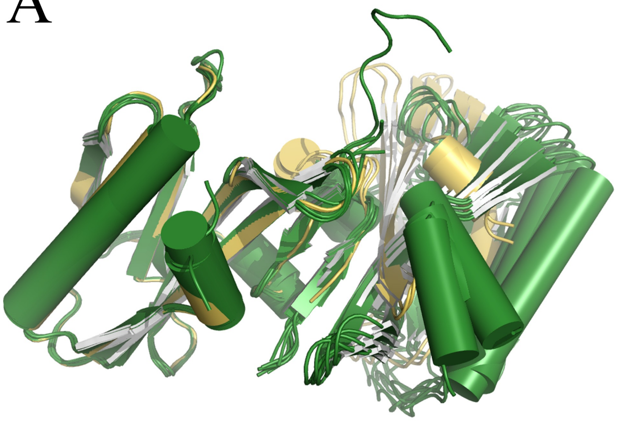

B

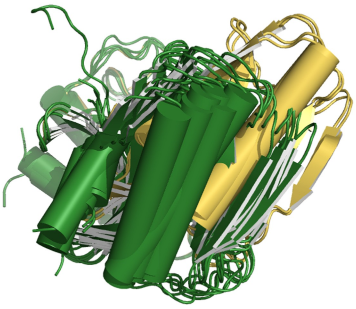

Supplementary Figure 1: Variability of NS1 ED strand-strand interactions. (A) Superposition of all NS1 ED strand-strand dimers aligned via the left-hand monomer of each pair to show the two distinct orientations observed. Dimers from 2GX9, 3O9Q, 3O9R, 3RVC, and two dimers from 3O9U (C and H, and E and G) are shown in dark green. Dimers formed by the A and F, and B and D chains of 3O9U adopt a different orientation, which is coloured gold. (B) Same superposition, viewed after a 90° rotation about a vertical axis.

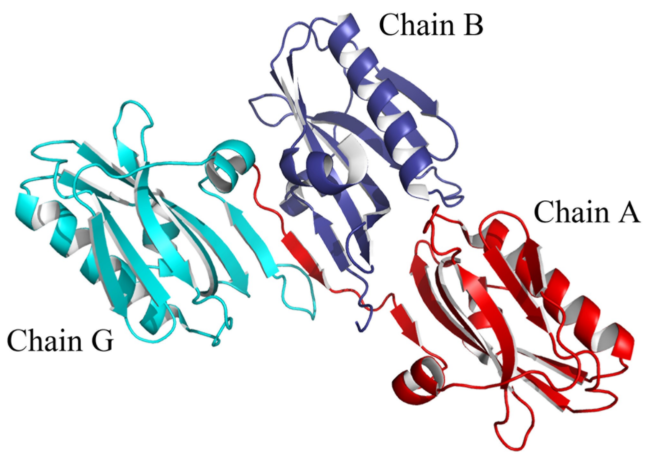

Supplementary Figure 2: Alternative β -strand addition interactions in the structure of an NS1 ED from A/California/07/09. Chains A, B and G of 3M5R are shown in red, blue and cyan, respectively.
